# Placental Circulation Intact Trial (PCI-T)—Resuscitation With the Placental Circulation Intact vs. Cord Milking for Very Preterm Infants: A Feasibility Study

**DOI:** 10.3389/fped.2018.00364

**Published:** 2018-11-27

**Authors:** Simone Pratesi, Simona Montano, Stefano Ghirardello, Fabio Mosca, Luca Boni, Lorenzo Tofani, Carlo Dani

**Affiliations:** ^1^Division of Neonatology, Careggi University Hospital of Florence, Florence, Italy; ^2^Neonatal Intensive Care Unit, Department of Mother and Infant Science, Fondazione IRCCS “Ca' Granda” Ospedale Maggiore Policlinico, University of Milan, Milan, Italy; ^3^Department of Human Pathology and Oncology, Clinical Trials Coordinating Center, Istituto Toscano Tumori, University of Florence, Florence, Italy; ^4^Department of Neuroscience, Psychology, Drug Research and Child Health, Careggi University Hospital of Florence, Florence, Italy

**Keywords:** randomized trial, preterm birth, delayed cord clamping, neonatal care at the bedside, cord milking, placental circulation intact

## Abstract

**Background:** Preterm newborns receiving briefly delayed cord clamping or cord milking at birth have better neonatal outcomes. However, the time frame in which both these procedures are performed (< 60 s of life) is too short to explore the possible beneficial effects on early infant postnatal adaptation and outcomes of a prolonged transfusion strategy associated with neonatal respiration.

**Methods and Design:** We have designed a randomized, multicenter, controlled two-phase study: phase 1 to assess the feasibility of carrying out the protocol in a large randomized trial, and phase 2 to assess the efficacy of bedside assistance with intact placental circulation for 3 min in comparison to cord milking to improve outcome in the neonatal period; we present here the feasibility and safety phase of the study. Outcomes included feasibility (recruitment rate of two patients per month, compliance with the trial interventions, completeness of data collection, >90% of infants receiving echographic assessments in the first 24 h) and safety variables (5 min Apgar score, delivery room intubation rate, CRIB II score, admission temperature, maximum hemoglobin concentration and hematocrit in the first 24 h and maximum serum bilirubin value) in the two study groups. We also evaluated the same safety variables in infants delivered during the study period but not recruited.

**Results:** A total of 40 infants were enrolled. In all cases the protocol was completed and all feasibility outcomes were reached. Infants assisted with an intact placental circulation have a higher 5 min Apgar score but their admission temperature was lower than milked infants. Delivery room intubation rate, CRIB II score and peak serum bilirubin value were comparable in both groups. Infants who were not subjected to a placental transfusion strategy (excluded patients) had a higher delivery room intubation rate with respect to both study groups.

**Conclusion:** Delaying cord clamping until 3 min of life was challenging but feasible and appeared to be safe. However, admission temperature must be strictly monitored and a more efficacious warming system could be implemented to prevent hypothermia during the procedure.

**Trial Registration:** Clinicaltrials,gov NCT02671305 (date of registration: 26 JAN 2016). https://clinicaltrials.gov/ct2/show/NCT02671305

## Introduction

If the umbilical cord is not clamped immediately after birth a significant volume of blood coming from the placenta passes into the newborn (1). Recent studies in animal models suggest a physiological role of placental transfusion (PT) in the first minutes of life and during the postnatal transition phase. By delaying cord clamping till after the start of efficacious breathing, heart left ventricle pre-load remains unchanged and major fluctuations in cerebral blood flow and pressures are prevented, possibly reducing the risk of cerebral vasculature rupture (2–5).

A recent meta-analysis demonstrates that delayed cord clamping is associated with improvement of cardiovascular stability in preterm infants and decreases the need for inotropes, the risk of intraventricular hemorrhage (IVH), necrotizing enterocolitis (NEC), oxygen requirement at 36 weeks of post-menstrual age, and blood transfusions without adverse effects (6).

PT in preterm infants might be slower than in term infants, and might be partial when the cord clamping is only briefly delayed, such as between 30 and 90 s (7). This is rational since at term pregnancy two-thirds of the feto-placental circulation is in the fetus, whilst at < 30 weeks gestation feto-placental circulation is mainly in the placenta (8). Moreover, the umbilical vein is smaller and uterine contractions are less efficient in preterm than in term pregnancies. Therefore, tiny preterm infants who start breathing during a prolonged PT might receive greater benefits and improve their outcomes. A recently updated systematic review including 19 trials comparing delayed vs. immediate cord clamping in preterm infants < 37 weeks gestation found that delayed clamping reduced hospital mortality (9).

Cord milking (MLK) has been suggested as a quick method to obtain a rapid PT in preterm births (10) allowing a timely resuscitation of the newborn if needed. MLK overrides the physiological control of infants' blood volume and blood pressure, disrupts umbilical blood flow and does not stimulate an increase in pulmonary blood flow (11, 12). However, in a recent meta-analysis it was found to decrease the risk of oxygen dependence at 36 weeks gestation and IVH in comparison to immediate cord clamping (13). Thus, relying on recently published results favoring MLK in preterm babies, we considered it more ethical that infants in the control group receive MLK instead of immediate cord clamping.

On the basis of these considerations, we hypothesized that clamping the cord after 3 min of life might be more beneficial than MLK in preterm infants < 30 weeks gestation, as the start of breathing/ventilating with placental circulation intact could favor a smoother and more physiological postnatal transition phase.

The purpose of this study is to assess the feasibility and safety of the protocol for a large multicenter trial.

## Methods

### Protocol design

A training phase preceded patient recruitment, with simulations performed on manikins and also on healthy term newborns from vaginal and cesarean deliveries.

Eligible infants were randomly assigned to either the bedside assistance with intact placental circulation group (PCI group) or the control group (cord milking) in a 1:1 ratio according to a computer-generated, randomized sequence. Randomization was stratified by gestational age (23+0 to 26+6 weeks or 27+0 to 29+6 weeks). The randomized allocation was concealed in double-enclosed, opaque, sealed, and sequentially numbered envelopes. The next sequential randomization envelope was opened only when the neonate was considered to be eligible by the attending operator.

All infants were resuscitated following the current guidelines of the American Academy of Pediatrics (AAP) (14). Newborns were positioned supine, covered with a plastic bag/wrap up to the shoulders without drying and the head immediately covered with a cap. Infants in the MLK group received at birth MLK without placental refill (milking 20 cm of cord four times at a speed of about 10 cm/sec), clamped within 20 s of life, and then assisted on a standard radiant infant warmer with the power output set at maximum. Infants in the PCI group were assisted at birth with placental circulation intact for the first 3 min of life on a portable resuscitation trolley (Lifestart trolley™, Inspiration Healthcare, UK) equipped with a warming mattress (CosyTherm, Inspiration Healthcare, UK) set at maximum temperature (40°C), a pulse oximeter (Radical-7, Masimo Corporation, USA), a suction system, a blender and flowmeter and a T-piece resuscitator (Neopuff Infant T-Piece Resuscitator, Fisher & Paykel, Auckland, New Zealand). Neonatal care was begun with FiO_2_ of 0.30 and initial peak inspiratory pressure (PIP) and positive end expiratory pressure (PEEP) values set at 20 and 5 cmH_2_O, respectively, in both groups. The flow rate was set at 10 L/min without changes during the resuscitation. Spontaneously breathing babies with a respiratory effort present or with a suboptimal SpO_2_ on pulse oximeter were assisted in nasal continuous positive airway pressure (CPAP). In the delivery room, infants were intubated and mechanically ventilated if they did not reach the goal of 70% SpO_2_ by 5 min (15) and 85% by 10 min of life (16) with a heart rate >100 bpm, despite nasal CPAP at 5–8 cmH_2_O or nasal positive pressure ventilation (PPV, with PIP 20–25 cmH_2_O and PEEP 5 cmH_2_O).

In case of cesarean section, the trolley and all neonatal resuscitation equipment placed on it, which might have contact with the operative field, were covered with a transparent sterile coverage. Thus, the obstetrician's hands placed the newborn on a completely sterile trolley and then returned to the mother's abdomen, while the neonatologist began to assist the baby and efforts were made for non-sterile equipment to only touch the neonatal provider's hands and the baby's face (for aspiration if necessary, and to position the mask and starting nCPAP/PPV as necessary). Heart rate was assessed by auscultation and pulse oximeter. During the 3-min period of delayed cord clamping the prevalent procedure was assisting respiratory function with nCPAP/PPV until intubation, if necessary. Early surfactant administration was also performed if necessary in the delivery room, but not during the 3-min period of delayed cord clamping.

All newborns were subjected to continuous cerebral oxygenation monitoring by near infrared spectroscopy (NIRS), a functional echocardiographic assessment together with cerebral and mesenteric blood flow evaluation, and cerebral echography in the first 24 h of life. Maternal postpartum hemorrhage or infection were also assessed.

Clinical characteristics of infants in PCI and MLK groups (Table 1) have been described using mean values and standard deviation or frequencies and percentage. The safety variables (Table 2) have been analyzed using Student's *t*-test for parametric continuous variables, the Mann-Whitney *U* test for non-parametric continuous variables and the chi-square test for categorical variables. A two-sided *p* < 0.05 was considered statistically significant.

**Table 1:** Characteristics of the study population.

	**PCI (14)**	**MLK (24)**	**Excluded pts(88)**	***p***
GA (w)	27.1 ± 1.3	26.7 ± 1.7	26.3 ± 2
BW (g)	955 ± 211 (500–1,300)	960 ± 305 (450–1,650)	903 ± 291 (430–1,679)
23–26 weeks (%)	28.5	29.1	43.1
Any prenatal steroid (%)	92.8	91.6	90.9
Cesarean Section (%)	42.8	54.1	56.8
Twins (%)	0^a^	0^a^	38.6^a^	< 0.002

*PCI, placental circulation intact group; MLK, milking group*.

*Data are presented as mean ± SD, or percentages*.

a *Statistical significance with p < 0.002 refers to Excluded patients vs PCI vs MLK*.

**Table 2:** Safety variables of study population and excluded patients.

	**PCI (14)**	**MLK (24)**	**Excluded pts (88)**	***p***
Median Apgar 5 min (IQR)	8 (7–8)^a^	7 (6–8)^a^	8 (7–8)	< 0.007
Body temperature on admission°C	35.5 ± 0.7^b^^c^	36.0 ± 0.5^b^	35.9 ± 0.7^c^	< 0.01^b^ < 0.02^c^
DR intubation rate (%)	14.2	12.5	34
Median CRIB II score (IQR)	8 (7–10)	9 (8–12)	9 (5–18)
Max Hb value first 24 h (g/dL)	20.4 ± 2.6^d^^e^	18.4 ± 2.9^d^	18.5 ± 3^e^	< 0.03^d^ < 0.01^e^
Max Ht value first 24 h (%)	62.5 ± 8^d^^e^	56.4 ± 9^d^	56.6 ± 9.6^e^	< 0.03^d^ < 0.01^e^
Max Bilirubin value (mg/dL)	8.5 ± 2.1	8.9 ± 2	8.6 ± 2.4

*PCI, placental circulation intact group; MLK, milking group*.

*Data are presented as mean ± SD; median, IQR (25th, 75th IQR) and percentages*.

a *Statistical significance with p < 0.007 refers to PCI vs MLK*.

b *Statistical significance with p < 0.01 refers to PCI vs MLK*.

c *Statistical significance with p < 0.02 refers to PCI vs Excluded patients*.

d *Statistical significance with p < 0.03 refers to PCI vs MLK*.

e *Statistical significance with p < 0.01 refers to PCI vs Excluded patients*.

### Inclusion and exclusion criteria and consent

The study protocol was carried out in accordance with the principles of the Declaration of Helsinki and approved by the Pediatric Ethical Committee of Tuscany.

Pregnant women admitted to the obstetrical unit and at risk of delivering between 23 0/7 and 29 6/7 weeks gestation were screened for participation. Written and oral information were offered to parents, and sufficient time was allowed for consent. Non Italian-speaking parents were only asked for their consent if an adult interpreter was available. The newborn entered the study only after both parents or legal representatives signed the informed consent form. Eligibility and consent were checked before randomization, which took place close to the time of birth, during labor or before cesarean section. A senior investigator was always available to discuss concerns raised by parents or clinicians during the study.

Infants whose parents consented underwent the protocol if delivery occurred between 23 0/7 and 29 6/7 weeks gestation without any of the following exclusion criteria: major congenital malformations and disorders, hydrops fetalis, placental or cord problems, or twin pregnancy.

### Outcomes

Outcomes included feasibility (recruitment rate of at least two patients per month, compliance with the trial interventions, completeness of data collection, >90% of infants receiving echographic assessments in the first 24 h of life) and safety variables (5 min Apgar score, delivery room intubation rate, CRIB score, admission temperature, maximum hemoglobin concentration and hematocrit in the first 24 h and maximum serum bilirubin value) in the two study groups. We also evaluated the same safety variables in infants delivered during the study period who were not included in the study and received immediate cord clamping.

## Results

Between April 2016 and March 2017 women expected to deliver at < 30 weeks GA were approached for consent. A total of 126 infants were eligible for participation in the study and the protocol was performed in 40 births: 20 infants assisted at mother's bedside during a 3-min delay in cord clamping, and 20 infants assisted after milking procedure followed by an early cord clamping (see Appendix). Only 49% of the 88 excluded infants met the exclusion criteria, while the other 51% were not enrolled for “organizational” problems (i.e., precipitous or emergency delivery, medical doctor refused or was unaware of the study, linguistic incomprehension). The characteristics of the study population and of excluded patients are reported in Table 1.

Six infants allocated to PCI assistance (30%) did not undergo the assigned procedure and received milking (four patients) or immediate cord clamping (two patients excluded post-randomization), as they were delivered precipitously without sufficient time for equipment setup or the cord was judged too short to perform neonatal assistance safely (in the four milked infants), or providers were unaware of the study (in the two infants immediately cord clamped, excluded post-randomization). All infants assisted with intact placental circulation were clamped at 3 min, except for one who was clamped after only 40 s as the provider judged the cord too short to perform assistance safely.

All feasibility outcomes were reached: recruitment rate of at least two patients per month in the final 6 months, efficacious neonatal assistance at mother's bedside during the 3-min delay in cord clamping (mouth and nose aspiration, positive pressure ventilation with T-piece or endotracheal tube were performed), completeness of data collection indicative of simple collection forms, and 100% of infants receiving echographic assessments in the first 24 h of life.

Infants assisted with intact placental circulation have a higher 5 min Apgar score, hemoglobin concentration and hematocrit in the first 24 h, but their admission temperature was lower than milked infants. Delivery room intubation rate, CRIB II score and peak serum bilirubin value were comparable in both study groups. Infants who did not receive a transfusion strategy (excluded patients) had a higher delivery room intubation rate with respect to both study groups (Table 2).

Maternal postpartum hemorrhage or infection rates were not different between groups. Maternal spinal anesthesia did not alter postnatal adaptation of infants born from cesarean section in both groups. None of the infants was born after general maternal anesthesia.

## Discussion

Delayed cord clamping after the start of breathing/ventilation could allow a gradual hemodynamic and respiratory post-natal adaptation (17). Newborns who start breathing or ventilating before cord clamping have better outcomes than newborns who start breathing or ventilating after cord clamping (18). Katheria et al. evaluated the ventilation effects during PT in infants with < 32 weeks gestation, performing cord clamping at 60 s of life (19). These researchers were not able to demonstrate a beneficial effect of ventilation, but about 90% of infants in their treatment and control groups breathed spontaneously during the first minute of life (19). In the more recent UK Cord Trial, the authors concluded that immediately assisting preterm infants with intact cord and delaying clamping until at least 2 min of life is feasible (20). In the large Australian placental transfusion study (APTS), preterm infants < 30 weeks gestation, whose cord was clamped at 1 min of life and received no neonatal assistance with intact cord, showed no differences in the composite outcome of death, severe brain injury, late-onset sepsis, NEC, or severe ROP compared to infants who received immediate cord clamping (< 10 s of life) (21).

In our study we found that assisting very preterm newborns with an intact cord during the first 3 min of life is feasible and safe. However, there are two main points limiting feasibility: the high percentage (51%) of eligible newborns not enrolled due to “organizational” problems, and the high percentage (30%) of protocol deviation in the PCI group. All causes of missing enrolment presented at the beginning of the study period were progressively resolved with no failures in the last months of the study period. Specifically, it was crucial to approach mothers for consent in a timely fashion and to constantly inform all providers for enrolment and randomization. Two physicians strictly monitored everyday admissions in the obstetrics ward and approached every eligible woman for consent. The signed consent was inserted in the mother's medical record and an updated list of women who consented to the study was available in the neonatology unit and delivery room. Protocol deviation in the PCI group was due to a relative shortness of the cord (2), precipitous delivery (2), and providers not aware of mother already randomized in the study (2). These problems were progressively resolved by (a) increasing neonatal providers' experience using the Lifestart trolley; (b) improving coordination between obstetrical and neonatal teams to obtain an optimal positioning of the trolley as close as possible to the mother's introitus or abdomen; and (c) informing all providers by email about mothers approached for consent. We also decided to purchase a second Lifestart trolley to reduce episodes of protocol failures due to precipitous deliveries with insufficient time to move the device from one room to another. One device is set up and ready to use in the operating room. In fact, the most challenging setting was during cesarean section because of the need for sterility and a more complicated positioning of the trolley near the abdomen of the mother.

Our two neonatal providers' (one doctor and one nurse) approach was proven effective in performing the first steps of neonatal resuscitation until intubation with an intact cord. However, the average admission temperature on arrival to the NICU in infants assisted at bedside for 3 min was lower than milked or immediately cord-clamped (excluded) infants. This is an important safety issue as neonatal hypothermia is associated with an increased neonatal morbidity in a dose-effect manner (14). Infants assisted at birth with placental circulation intact lay supine on a neonatal warming mattress, which starts warming only when the newborn is placed on it. For this reason, the mattress could be less efficient in lighter babies (weighing < 1,000 grams). However, milked and immediately-clamped newborns were also hypothermic in our study. Furthermore, preterm infants immediately assisted on the same device with intact cord were not hypothermic at admission to NICU (20). We found that delivery room temperature was a critical point in our center (22–24°C), being less than that recommended for very preterm deliveries. On the basis of these findings, we have recently made arrangements to increase delivery room temperature, obtaining a temperature of 26°C in the neonatal resuscitation area and 24–25°C in the delivery room where preterm newborns are spontaneously delivered. However, the use of a more efficient warming mattress could be necessary in lighter newborns during the PCI procedure (above all in the operating room).

Newborns assisted without clamping the cord for 3 min (PCI group) had a higher 5-min Apgar score than milked newborns, demonstrating that it is possible to perform neonatal assistance at mother's bedside effectively, perhaps with a better early postnatal adaptation, and they had a higher hemoglobin concentration and hematocrit in the first 24 h, possibly due to a higher volume of placental blood transfused at birth. Increased placental transfusion in PCI infants could be due to a longer period of unclamped cord during which the newborns' lungs are ventilated.

## Conclusion and future directions

Delaying cord clamping until 3 min of life while assisting the baby at the mother's bedside was challenging but feasible and appeared to be safe in this study. However, delivery room and admission temperature of the newborn must be strictly monitored to evaluate the efficacy of the warming mattress used.

A phase 2 randomized multicenter clinical study is underway to assess the efficacy of bedside assistance with intact placental circulation for 3 min in comparison to cord milking as a way to improve outcome in the neonatal period.

## Data availability statement

The datasets during and/or analyzed during the current study will be available from the corresponding author upon reasonable request.

## Author contributions

SP, SG, CD, and FM contributed to conception and design of the study. LB and LT organized the database. LB and LT performed the statistical analysis. SP wrote the first draft of the manuscript. SP and SM wrote sections of the manuscript. All authors contributed to manuscript revision, read and approved the submitted version.

### Conflict of interest statement

The authors declare that the research was conducted in the absence of any commercial or financial relationships that could be construed as a potential conflict of interest.

## Figures and Tables

**Figure A1:**
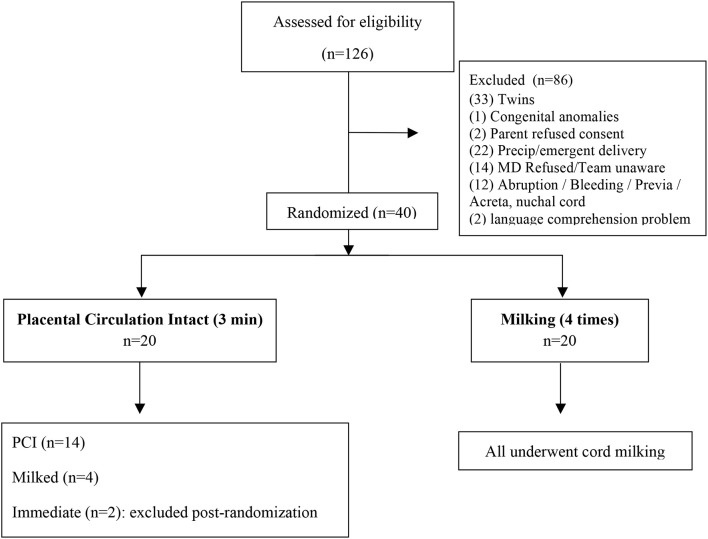
CONSORT diagram: study enrollment, intended randomization, and actual randomization.
